# Vitamin B12 Levels in Methamphetamine Addicts

**DOI:** 10.3389/fnbeh.2018.00320

**Published:** 2018-12-18

**Authors:** Changping Zhai, Ming Cui, Xiaodong Cheng, Xiang Ao, Tingting Zhao, Wei Wu, Qun Shao, Dexue Ge, Hongmei Song, Fangzhi Qi, Qiang Ling, Mengdi Ma, Mengyuan Xu, Dongliang Jiao

**Affiliations:** ^1^Anhui Province Veterans Hospital, Bengbu, China; ^2^Compulsory Isolated Drug Rehabilitation Center, Bengbu, China; ^3^Department of Psychiatry, Bengbu Medical College, Bengbu, China

**Keywords:** methamphetamine, addiction, neurotoxicity, vitamin B12, homocysteine

## Abstract

**Objective:** It has been established that reduced vitamin B12 serum levels are associated with cognitive decline and mental illness. The chronic use of methamphetamine (MA), which is a highly addictive drug, can induce cognitive impairment and psychopathological symptoms. There are few studies addressing the association of MA with vitamin B12 serum levels. This study examined whether the serum levels of B12 are associated with MA addiction.

**Methods:** Serum vitamin B12, homocysteine (Hcy), glucose and triglyceride concentrations were measured in 123 MA addicts and 108 controls. In addition, data were collected on their age, marital status, level of education and Body Mass Index (BMI) for all participants. In the patient group, the data for each subject were collected using the Fagerstrom Test for Nicotine Dependence (FTND), the Alcohol Use Disorders Identification Test (AUDIT), and a drug use history, which included the age of onset, total duration of MA use, the number of relapses and addiction severity.

**Results:** Our results showed that MA addicts had lower vitamin B12 levels (*p* < 0.05) than those of healthy controls, but Hcy levels were not significantly different between the two groups (*p* > 0.05). Serum B12 levels were negatively correlated with the number of relapses in the MA group. Furthermore, binary logistics regression analysis indicated that the B12 was an influencing factor contributing to addiction severity.

**Conclusion:** The findings of this study suggest that some MA addicts might have vitamin B12 deficiency, and serum B12 levels may be involved in the prognosis of MA addiction.

## Introduction

The problem of addiction to amphetamine-type stimulants (ATS) has become a global public health problem. The most common ATS is methamphetamine (MA). MA is highly addictive and can lead to psychiatric illnesses or cognitive defects(Curran et al., 2004; Scott et al., 2007; Jacobs et al., 2008). Psychotic symptoms induced by ATS include hallucinations, delusions (Bramness et al., 2012), depression (Sutcliffe et al., 2009), and anxiety(Bagheri et al., 2015). A strong relationship between cognitive defects or psychotic symptoms and substance abuse relapse or prognosis has been demonstrated in laboratory and clinical studies; treatment of cognitive and mental disorders may contribute to the prognosis of addiction (Liu et al., 2016). However, the mechanism of cognitive dysfunction and psychiatric illnesses induced by MA use has not been fully explained, and there has been a lack of effective medicines to treat ATS-related problems.

It has been proposed that a decreased B12 level is responsible for neuropsychiatric disorders (Kumar, 2014). For example, B12 levels were lower (Kim et al., 2008; Saedisomeolia et al., 2011; Almeida et al., 2015) in patients with schizophrenia and affective disorders compared to those in healthy individuals. Reduced B12 levels may affect the brain through multiple mechanisms and are involved in the occurrence of disease (Tangney et al., 2011). There is a great deal of evidence suggesting that vitamin B12 supplementation may be important for treating certain patients (Moore et al., 2012; Brown and Roffman, 2014). However, there have never been any reports examining whether serum levels of B12 are associated with ATS addiction.

The causes of B12 deficiency are multifactorial, including nutritional causes due to poor dietary intake, as well as functional causes due to impairments in absorption and intracellular processing and trafficking. Vitamin B12 is a vitamin that requires an intestinal secretion (endogenous factor) to be absorbed. Therefore, conditions associated with malabsorption, such as ileal disease or resection, may cause B12 deficiency (Carmel, 2000). MA can induce intestinal disease (Beyer et al., 1991; Herr and Caravati, 1991; Brannan et al., 2004; Prendergast et al., 2014). Thus, chronic long-term abuse of MA may cause nutrient absorption disorders. Recently, it has been reported that B12 may alleviate MA-induced neurotoxicity (Moshiri et al., 2018). Therefore, we speculate that MA abuse may affect the absorption of B12 and lead to B12 deficiency.

Homocysteine (Hcy) is a thiol group-containing amino acid that naturally occurs in all humans. An elevated level of Hcy can often be caused by deficiencies in vitamin B12. Elevated Hcy could induce oxidative stress (Papatheodorou and Weiss, 2007), lipid metabolism imbalance (Liao et al., 2006), N-methyl-d-aspartate receptor overexcitation (Kim and Pae, 1996), tau hyperphosphorylation and accumulation (Wei et al., 2011) and upregulation of hepcidin (Luo et al., 2016), which may lead to cognitive deficits, such as Alzheimer's disease (Xu et al., 2012; Castillo-Carranza et al., 2017; Sternberg et al., 2017; Wang et al., 2017) and psychiatric disorders (Osher et al., 2008; Kinoshita et al., 2013). Homocysteine has been a potential predictor for dementia (Zou et al., 2018). Thus, we hypothesized that B12 and Hcy may be involved in MA addiction-related problems.

The aim of the present study was to examine (1) whether serum levels of B12 and Hcy in MA-dependent patients are different from healthy controls and (2) whether there is any relationship between MA addiction severity and the levels of B12 and Hcy.

## Methods

### Participants

A total of 123 male MA addicts was recruited from the Compulsory Isolated Drug Rehabilitation Center in Bengbu. The MA addicts who participated in this study met the diagnosis of MA dependence according to the Diagnostic and Statistical Manual of Mental Disorders (DSM-V) criteria. The diagnoses were verified by a senior psychiatrist with the rank of associate professor.

The control group (108 men) was recruited from staff members of the Bengbu Mental Health Center and the local community, and none of them had any history of drug use. The controls were matched with the MA groups in terms of gender, age and education. All subjects in the study participated voluntarily and signed an informed consent form for the protocol. The study was approved by the Institutional Review Board (permission number: 2017-53) of Bengbu Medical University. All experiments were carried out in accordance with the approved guidelines and regulations.

Blood samples were collected on the first day when the participants came into the center, and the relevant indicators were tested on the same day. The inclusion criteria were (1) no history of severe mental illness, (2) age between 18 and 45 years old, (3) normal vision and hearing, (4) more than 9 years of education, and (5) abstinent from MA for <3 months.

The exclusion criteria were (1) psychiatric or neurological illness (e.g., schizophrenia, affective disorders, stroke, seizure, or parkinsonism); (2) other chronic diseases (e.g., diabetes, hypertension, hyperlipemia, and gastroenteropathy); (3) other substance dependence (e.g., opioids and cocaine), except for cigarettes and alcohol, within the past 5 years; and (4) being a vegetarian.

### Clinical and Neuropsychological Assessment

Before the tests, detailed questionnaires, including a complete medical history, physical and psychological examination, were obtained from each subject.

All participants filled out a self-administered case report form including their age, marital status, and level of education. In the patient group, each subject was interviewed by one psychiatrist, and data were collected on the Fagerstrom Test for Nicotine Dependence (FTND), the Alcohol Use Disorders Identification Test (AUDIT), and a drug use history, which included the age of onset, total duration of MA use, the number of relapses, etc. Addiction severity was assessed using a self-administered case report form that included questions about subjective craving feelings and the degree of drug dependence. Simultaneous Body Mass Index (BMI = weight/height squared) was measured in the field.

### Assessment of Serum B12, Hcy, Glucose, and Triglyceride Levels

Fasting blood samples were collected from each participant between 7:00 and 9:00 a.m. (before breakfast) for the measurement of 4 markers, including B12, Hcy, glucose and triglyceride. Serum concentrations of these markers were measured 3 h later by a technician who was blinded to the diagnostic status of the subjects. Hcy, glucose and triglyceride levels were measured using a HITACHI 7600 automatic biochemistry analyzer (Hitachi High-Technologies Corporation, Japan), and the B12 levels were measured using a MAGLUMI 2000 automatic chemiluminescence immunoassay analyzer (New Industries Biomedical Engineering Co., Ltd., Shenzhen, China).

### Statistical Analysis

The data were analyzed using SPSS, version 16.0 (SPSS Inc., an IBM Company Headquarters, 233 S. Wacker Drive, 11th floor Chicago, Illinois 60606). Group differences were compared using the Student's *t*-test for continuous variables, the nonparametric Mann-Whitney *U*-test for abnormal distribution variables and the chi-squared test for categorical variables. The correlation between variables was studied using Spearman's correlation. A binary logistics regression analysis was done with addiction severity as the dependent factor and age, education, age of onset, total duration of MA use, number of relapses, AUDIT score, FTND score, B12, and Hcy as independent factors. The alpha level was reported with *p* < 0.05 (two-sided tests) considered to be statistically significant.

## Results

### Demographics and Drug Use History Data (Table 1)

There were no differences in age, marital status, BMI, glucose or triglyceride between the two groups (*p* > 0.05). The MA group had fewer years of education. Information on smoking and alcohol consumption in the controls was unavailable. The MA group results had either quartiles or a mean for FTND score (1.00, 3.00, 6.00), AUDIT score (0.00, 3.00, 13.50), age of onset (years) (26.24 ± 7.61), the number of relapses (1.00, 1.00, 2.00), total duration of MA use (months) (3.00, 12.00, 36.00), and addiction severity (0.00, 3.00, 6.00).

**Table 1:** Demographics and drug use history data, quartile (25,50,75) or Mean ± SD (standard deviation).

**Variable**	**MA group (*n* = 123)**	**HC group(*n* = 108)**	**F/Z/x^**2**^**	***p***
Age (years) (mean ± SD)	34.20 ± 7.99	35.75 ± 12.28	22.40	0.27
Years of education (years) (25,50,75)	1.00, 2.00, 2.00	2.00, 5.00, 6.00	−10.18	0.00
Married (%)	96 (78.0%)	80 (74.0%)	0.50	0.54
BMI (mean ± SD)	24.07 ± 3.96	24.02 ± 2.54	0.09	0.93
Glucose (25,50,75)	4.56, 4.99, 5.88	4.91, 5.12, 5,50	−1.42	0.16
Triglyceride (25,50,75)	0.80, 1.16, 1.73	0.96, 1.35, 2.07	−1.76	0.08
FTND score (25,50,75)	1.00, 3.00, 6.00			
AUDIT score (25,50,75)	0.00, 3.00, 13.50			
Age of onset (years) (mean ± SD)	26.24 ± 7.61			
The numbers of relapse (25,50,75)	1.00, 1.00, 2.00			
Total duration of MA use (month) (25,50,75)	3.00, 12.00, 36.00			
Addiction severity (25,50,75)	0.00, 3.00, 6.00			

*FTND, Fagerstrom Test for Nicotine Dependence; AUDIT, The Alcohol Use Disorders Identification Test; BMI, Body Mass Index*.

### Vitamin B12 and Hcy Levels in MA and Healthy Controls (Table 2)

The quartiles for vitamin B12 levels were (224.70, 328.00, 424.70) pg/mL in the MA group and (341.4, 390.1, 542.80) pg/mL in the control group. Vitamin B12 levels were significantly lower in the MA group than in healthy controls (*Z* = −5.37, *p* = 0.000). Hcy levels were not significantly different between the two groups (*p* > 0.05).

**Table 2:** Plasma vitamin B12, Hcy levels in MA and healthy controls.

	**MA group(*n* = 123) (25, 50, 75)**	**HC group (*n* = 108) (25, 50, 75)**	***z***	***p***
B12 (pg/mL)	224.70, 328.00, 424.70	341.4, 390.1, 542.80	−5.37	0.00
Hcy (μmoi/L)	10.80, 14.20, 24.30	12.50, 14.20, 17.40	−0.33	0.74

### The Association Between B12 and Hcy Levels With Drug Use History, FTND Score and AUDIT Score in the MA Group

**Figure 1:**
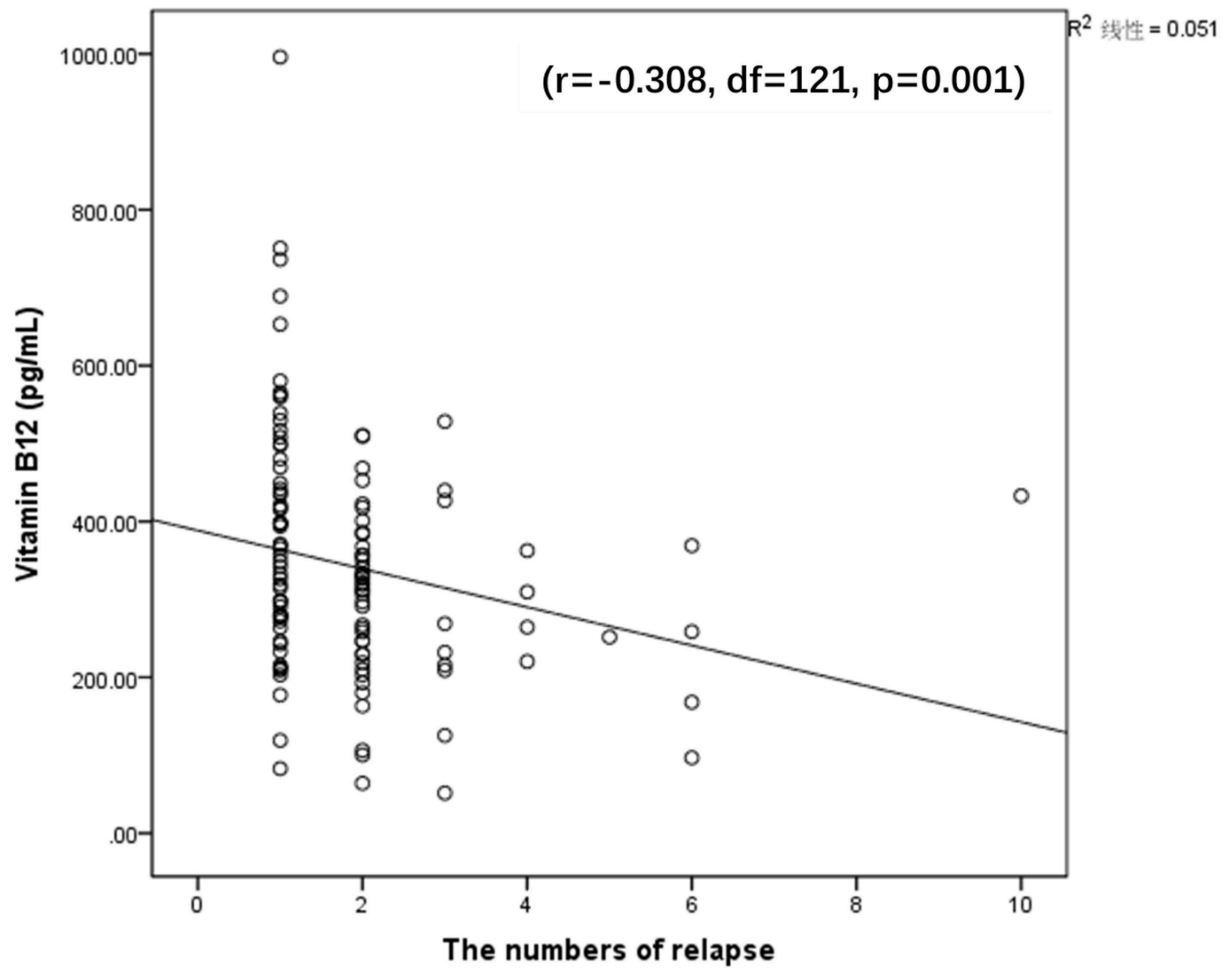
Significantly negative correlation between vitamin B12 and the number of relapse (*r* = −0.308, df = 121, *p* = 0.001).

A significant, negative correlation was observed between B12 and the number of relapses (*r* = −0.308, df = 121, *p* = 0.001) (Figure 1) in the MA group. There was no significant correlation between Hcy levels and drug use history in the MA group (all *p* > 0.05). There was no significant correlation between B12 and Hcy levels with the FTND or AUDIT scores in the MA group. These results suggest that the more relapses an individual had, the more significant the impact was on the decline of B12.

### Influencing Factors on Addiction Severity

FTND, AUDIT, age of onset, the number of relapses, total duration of MA use, B12 and Hcy were taken as independent variables, and the addiction severity was taken as a dependent variable for binary logistic regression analysis. The obtained logistic model had statistical significance, $x_{\left(7\right)}^{2}$ = 17.176, *P* = 0.016. B12 (beta = −0.006, Exp_(B)_ = 0.994, *p* = 0.038) and FTND (beta = 0.220, Exp_(B)_ = 1.246, *p* = 0.036) were statistically significant among the seven independent variables (or predictors) included in the model.

## Discussion

The major findings of this study were that (1) B12 levels were significantly reduced in MA-dependent patients compared the healthy controls, (2) the decrease in B12 was correlated with the number of relapses, and (3) the decrease in B12 was a factor influencing the severity of drug addiction.

To the best of our knowledge, this study was the first to find that serum B12 levels were significantly lower in MA-dependent patients compared to those in healthy controls. B12 is absorbed from the intestine, and it is a vitamin that requires an intestinal secretion (intrinsic factor) to be absorbed. It is affected by intestinal function (Lima et al., 2018); therefore, intestinal diseases may lead to B12 absorption disorders. MA could cause splanchnic vasoconstriction and necrotizing angiitis (Johnson and Berenson, 1991; Kelly et al., 1992), which can even induce intestinal ischemia and infarction (Beyer et al., 1991; Herr and Caravati, 1991; Brannan et al., 2004; Prendergast et al., 2014). Thus, chronic long-term abuse of MA could cause nutrient absorption disorders, weight loss and malnutrition. For example, previous studies found that the serum folic acid in MA-dependent patients was significantly lower than in the controls (Nakazawa et al., 1981), and folic acid uptake was decreased by MA in primary cultures of human syncytiotrophoblasts (Keating et al., 2009). Although we did not test folic acid levels, we found that B12 levels were significantly decreased in MA-dependent patients; therefore, we speculated that the cause of decreased serum B12 levels might due to a disorder in B12 absorption caused by MA.

In addition, there was no significant difference in Hcy levels between the MA group and the control group in this study. Hcy is metabolized through two major pathways: transsulfuration and methylation. Approximately 50% is catabolized in the transsulfuration pathway, which does not require B12 (Bolander-Gouaille, 2002). The results suggest that Hcy may not be involved in the mental disorders and cognitive impairment induced by MA.

MA, as a nervous stimulant, can induce severe neurotoxicity by oxidative stress (Solhi et al., 2014; Zhang et al., 2018), damage to the blood-brain barrier (Northrop and Yamamoto, 2015) and overexpression of pro-inflammatory cytokines (Tocharus et al., 2010), which leads to subsequent psychiatric disorders or cognitive deficits (Curran et al., 2004). Vitamin B12 is essential for the development and initial myelination of the central nervous system, and vitamin B12 deficiency can lead to a wide range of neurological symptoms (Lövblad et al., 1997). A decrease in vitamin B12 may be involved in MA-induced neurotoxicity in the following ways. (1) Overexpression of pro-inflammatory cytokines. It was reported that a low B12 level was associated with overexpression of pro-inflammatory cytokines (Politis et al., 2010). Pro-inflammatory cytokines might be involved in the pathophysiology of many psychiatric conditions (Banerjee et al., 2017; Vakilian et al., 2018). (2) Mitochondrial DNA disorders. Vitamin B12 plays an important role in the maintenance of mitochondrial genome integrity (Fenech, 2012). B12 deficiency could induce mitochondrial DNA sequence variation and might contribute to cognitive function disorders and psychiatric symptoms (Inczedy-Farkas et al., 2012; Tranah et al., 2012; Zaia et al., 2017). (3) Promoting oxidative stress. Vitamin B12 deficiency could also induce neurotoxicity by promoting oxidative stress (Misra et al., 2016; Zhang et al., 2016; Bito et al., 2017). (4) Damaging the blood-brain barrier. Blood—brain barrier pathology is recognized as a central factor in the development of many neurological disorders. Experimental and human evidence support the idea that vitamin B12 plays a role in maintaining the integrity of the blood—brain barrier (Lehmann et al., 2003; Pieters et al., 2009; Pollak et al., 2018). Neurotoxicity induced by B12 deficiency might contribute to various types of neuropsychiatric disorders (Bell et al., 1991; Türksoy et al., 2014; Zhang et al., 2016) and cognitive impairment (Wang et al., 2001; Tangney et al., 2011). In conclusion, Vitamin B12 deficiency might be involved in MA-induced neurotoxicity in these ways and induce mental disorders as well as cognitive impairment.

However, there is little research on how b12 was involved in the formation of MA addiction. According to previous research, we speculate that cognitive impairment and psychiatric disorders, which reflect damage to brain regions associated with addiction (Everitt et al., 2007), could exacerbate drug addiction (Chambers et al., 2009) and disrupt treatment and prognosis (Aharonovich et al., 2006; Witkiewitz and Bowen, 2010; Hellem, 2016). In addiction, it has been reported that decreased vitamin B12 availability induces ER stress (Ghemrawi et al., 2013). Our previous study (Jiao et al., 2017) showed that ER stress was involved in the process of MA addiction. Thus, decreased vitamin B12 might be directly involved in the formation of MA addiction through ER stress.

The current study suggests that B12 was associated with the number of relapses and is one of the important factors influencing the severity of addiction. These results suggest that a decrease of B12 may affect the prognosis of addiction. Recent research has reported (Moshiri et al., 2018) that B12 at safe doses may be a promising treatment for MA-induced brain damage through inhibition of neuron apoptosis and increasing the reduced GSH level. B12 could serve as a novel therapeutic agent against MA-induced neurotoxicity.

The shortcomings of this study were that participants were not tested for their cognitive and psychosis status, which is a major concern among addicts. Thus, it is necessary to perform additional studies to explore whether the decreased vitamin B12 levels correlate with cognitive dysfunction and psychosis in MA addicts. Some reports show that the consumption of cigarettes and wine correlates with decreased vitamin B12 (Seethalakshmi and Chinnaswamy, 2006; Haj Mouhamed et al., 2011; Fragasso, 2013). However, the present study did not find that the consumption of cigarettes and wine were correlated with plasma vitamin B12 in MA addicts; therefore, it is necessary to compare MA addicts with the healthy group to confirm the associations between these factors.

## Ethics Statement

All subjects in the study were voluntary and signed an informed consent for the protocol. The study was approved by the Institutional Review Board of Bengbu Medical University (permission number: 2017-53). All experimental procedures in this manuscript were in strict accordance with the approved guidelines and regulations.

## Author Contributions

CZ, MC, and DJ conceived and designed the experiments. CZ, TZ, WW, QS, DG, MM, and MX carried out experiments. XA, TZ, and HS analyzed experimental data. FQ, XC, and QL contributed reagents, materials, analysis tools. CZ and TZ wrote the first draft of the manuscript. DJ provided critical revision of the manuscript for important intellectual content.

### Conflict of Interest Statement

The authors declare that the research was conducted in the absence of any commercial or financial relationships that could be construed as a potential conflict of interest.
